# Laccase-Enzyme Treated Flax Fibre for Use in Natural Fibre Epoxy Composites

**DOI:** 10.3390/ma13204529

**Published:** 2020-10-13

**Authors:** Hanna M. Brodowsky, Anne Hennig, Michael Thomas Müller, Anett Werner, Serge Zhandarov, Uwe Gohs

**Affiliations:** 1 HTWK, Leipzig University of Applied Sciences, D-04277 Leipzig, Germany; 2 Formerly Leibniz Institute of Polymer Research (IPF), D-01069 Dresden, Germany; anne_hennig@protonmail.com; 3 Leibniz Institute of Polymer Research (IPF), D-01069 Dresden, Germany; mueller-michael@ipfdd.de; 4 Bioprocess Engineering, Institute of Natural Materials Technology, Faculty of Mechanical Science and Engineering, Technical University Dresden, D-01069 Dresden, Germany; anett.werner@tu-dresden.de; 5 V.A. Bely Metal-Polymer Research Institute of the National Academy of Sciences of Belarus, 246050 Gomel, Belarus; serge.zhandarov@gmail.com; 6 Institute of Lightweight Engineering and Polymer Technology, Faculty of Mechanical Science and Engineering, Technical University Dresden, D-01307 Dresden, Germany; Uwe.Gohs@tu-dresden.de

**Keywords:** natural fibre composites, enzyme, laccase, dopamine, interphase

## Abstract

Natural fibres have a high potential as reinforcement of polymer matrices, as they combine a high specific strength and modulus with sustainable production and reasonable prices. Modifying the fibre surface is a common method to increase the adhesion and thereby enhance the mechanical properties of composites. In this study, a novel sustainable surface treatment is presented: the fungal enzyme laccase was utilised with the aim of covalently binding the coupling agent dopamine to flax fibre surfaces. The goal is to improve the interfacial strength towards an epoxy matrix. SEM and AFM micrographs showed that the modification changes the surface morphology, indicating a deposition of dopamine on the surface. Fibre tensile tests, which were performed to check whether the fibre structure was damaged during the treatment, showed that no decrease in tensile strength or modulus occurred. Single fibre pullout tests showed a 30% increase in interfacial shear strength (IFSS) due to the laccase-mediated bonding of the coupling agent dopamine. These results demonstrate that a laccase + dopamine treatment modifies flax fibres sustainably and increases the interfacial strength towards epoxy.

## 1. Introduction

Fibre reinforced polymers combine the superior mechanical properties of fibres with the usability of a polymer. The interphase between fibre and matrix plays an essential role for the composites properties as it is crucial in transferring an external stress from the matrix to the reinforcing fibres. A fibre-reinforced composite can only be as strong as its interface. Conventional reinforcing fibres such as glass, aramid or carbon fibres are usually treated with a sizing or finish in order to improve the adhesion to the polymer matrix [1,2]. These sizings are aqueous solutions. They contain bi-functional coupling agents such as silanes, which ideally covalently bond to both fibre and matrix, as well as film formers, which ensure the processability of the fibre and may also aid in adhesion. Such sizings need to be optimised with the specific fibre–matrix combination in mind. In natural fibre composites, surface treatments are seldom used so far, even though they would increase the mechanical properties of the composites [3,4,5,6,7]. 

Natural fibres are a sustainable alternative to conventional fibres. With respect to specific strength and modulus, they are comparable to glass fibres [3]. Excellent damping properties make them attractive for applications such as car interiors or sports equipment. Reinforcing natural fibres for plastic matrices are usually bast fibres, obtained from, e.g., flax, jute or hemp. Flax, as used in this study, consists of roughly 71% cellulose, 18.6% to 20.6% hemicellulose, 2.3% pectine, 1.7% wax and 2.2% lignin [6,7]. Cellulose fibres form the load-bearing structure, with hemicellulose as support. Lignin interlinks those structures and is essential for increasing the overall fibre strength. The more lignin is present in a plant fibre, the more wood-like it becomes.

Various procedures for surface treatment of natural fibres to either increase their mechanical properties or fibre matrix adhesion have been reported, including mercerisation, alkali treatments, acetylation, copolymerisation and the use of silanes or triazines as coupling agents [5,6,8,9,10,11,12]. Products such as silanes are produced petrochemically and are hazardous towards land and water life forms and stay on the fibres throughout their life cycle, negating the natural fibre advantages of ecological production and degradation. 

To replace these often unecological surface modifications, the focus of this study is a sustainable enzymatic treatment of the flax fibre, using laccase from a cerrena unicolor polypore fungus, and dopamine as a coupling agent. Laccase (EC 1.10.3.2) is an enzyme of the group of oxidases found in fungi as well as plants, bacteria and animals. In combination with other enzymes, laccase is catalysing polymerisation and depolymerisation processes by oxidising substrates. Substrates specific for laccase are mainly phenols. Besides, using mediator molecules allows for a wider range of possible substrates. Mediators in this case are small organic compounds mediating between enzyme and substrate through either of three routes: electron-transfer, radical hydrogen transfer, or ionic oxidation [13,14,15,16,17,18,19,20].

In a natural process commonly known as whiterotting, lignin is degraded by the laccase enzyme via oxidation [21,22,23]. Natural laccase often works in combination with other enzymes such as lignin peroxidase. In [24], the authors concluded that the specific role of laccase lies in demethylation as well as activating mediators and radicals as support for the depolymerisation by lignin peroxidase. In the present study, using neat laccase should result in minimal damage to the lignin or the fibre. Laccase-activated phenoxy radicals should be able to react with other compounds. Laccase is abundant in nature and sustainable. Besides, it is available in sufficient quantities and robust enough to be used in numerous industrial processes, e.g., in the food industry for processing and stabilizing beverages and additives in baking [25,26], for decolourisation and degradation of dyes in the textile industry, for pollution control in water and soil [17,27,28,29,30] as well as for pulp bleaching in the pulp and paper industry [24,31,32].

In the past, laccases and other enzymes have already been reported to modify cellulosic structures. In [33,34], laccase has been used to increase the hydrophobicity of jute fibre by covalently grafting dodecyl gallate to the surface, with the aim to improve wetting behaviour towards a polypropylene matrix. Increasing the hydrophobicity of beech veneer by laccase-catalysed coupling of fluorophenols was carried out by Kudanga et al., increasing the contact angle by between 10% and 65%, depending on the fluorophenol used. The authors also identified covalent coupling between fluorophenols and the lignin model compounds guaiacylglycerol b-guaiacyl ether and syringylglycerol b-guaiacyl ether by NMR and LC-MS analysis [35]. Successful coupling of phenolic amines and fluorophenols to another lignin model compound, dibenzodioxocin, was reported as well [36]. In hemp [37] and abaca [38], laccase has been used to remove lignin from the fibre and increase the cellulose content. Herrero Acero et al. showed that laccase fibre modification can be used to bond phenol or amine moieties to the fibre surface [39]. In addition, enzyme blends have successfully been used to improve surface and thermal properties of both flax and hemp [40]. Kim and Cavaco-Paulo successfully used laccase to covalently bind flavonoid compounds to proteins for medical applications [41]. In sum, laccase has often been used to graft hydrophobic molecules to the fibre surface in order to adjust the surface energy, and thereby improve the adhesion. However, the state of the art in conventional fibre sizing is the use of bifunctional coupling agents, joining the fibre surface and matrix on the base of covalent bonds. The aim of the present study is to transfer this scheme to natural fibres, using the biological reactant dopamine as a bifunctional coupling agent in order to increase interfacial strength and improve the mechanical parameters of the composite. 

Dopamine is a small organic molecule, abundant in nature. It is a phenol derivative with an alkylamine substituent. On the one hand, dopamine should be able bind to the flax fibres’ lignin via laccase catalysis, on the other hand it may bind to the epoxy groups within cold-curing processes or to acid anhydrides within heat-curing processes. The phenolic functional groups of dopamine will be converted to phenoxy radicals by laccase modification in the same process as was discussed for lignin [42,43]. In combination, this should effectively connect the fibre and matrix covalently.

The focus of the present study is an enzymatic treatment of the flax fibre to replace silanes or other often unecological coupling agents. Flax fibres are treated with laccase and dopamine solution. During laccase treatment, the flax fibre mechanical properties are preserved. Proposedly, a layer of the coupling agent is evident in SEM and AFM micrographs. The interfacial shear strength (IFSS) is determined by micromechanical pullout measurements on flax fibre epoxy microcomposites. The laccase dopamine treatment improves the IFSS by 30%.

## 2. Materials and Methods

Flax fibre yarn (250 tex) was obtained from composites evolution (Chesterfield, UK). It was chosen as it contains mostly single fibres of 2–6-cm length and 20-µm diameter that could be used without further separation. Dopamine hydrochloride of TraceCert^®^ grade with a melting point at 248–250 °C and a molecular weight of 153.18 g/mol (without HCl) was obtained from Sigma (St. Louis, MO, USA). Epoxy resin/hardener system RIM 135/RIM 137 was obtained from Hexion (Columbus, OH, USA).

### 2.1. Laccase Treatment

Cerrena unicolor was purchased from Greentech Enzymes GmbH (Ballrechten-Dottingen, Germany). The organism was maintained on 2% (*w*/*v*) potato glucose agar plates at 4 °C. The preculture was cultivated for seven days at 150 rpm, 26 °C in a rotary shaker, model KS 4000 ic control (IKA, Staufen, Germany), before transferring 10% (*v*/*v*) of the preculture into the main culture containing the natural medium. Main cultures were cultivated in in bioreactors (total volume 5 L) with a working volume of 3 L. These were cultivated at 26 °C, with an agitation rate of n = 300 rpm and an aeration rate of 0.5 vvm. To prevent foam formation in the fermenter, an acid regulation was adjusted at pH 5.5, adding 2 M H_2_SO_4_ if the pH was exceeded. Cultures were harvested after the maximum laccase activity (240 U/mL) was exceeded. 

The natural medium adapted from [44] contained the following components per L: 0.8 g KH_2_PO_4_, 0.2 g K_2_HPO_4_, 0.5 g MgSO_4_∙7 H_2_O, 3 g peptone, 3 g yeast extract, 0.025 g CuSO_4_∙5 H_2_O, 20 g granulated orange peel and 20 g wheat bran.

Laccase activity was spectrophotometrically determined by the oxidation of 0.1 mM 2,2′-azino-bis(3-ethylbenzothiazoline-6-sulfonic acid) diammonium salt—ABTS—(Thermo Fisher Scientific, USA) in 0.1 M malonate buffer, pH 5 at 25 °C and 420 nm (ε420 = 36000 /M/cm). In duplicate, 10 µL of sample was suspended in 1000 µL of ABTS solution, the reaction was monitored for 60 s on a Beckman DU-640 spectrophotometer (Beckmann, Fullerton, CA, USA) and the laccase activity (U/mL) was calculated by the increase in absorption over time. One unit was defined as the amount of enzyme that catalyses the reaction of 1 µmol substrate per minute. 

Cultures were centrifuged multiple times at 15,000 g, 4 °C for 20 min (Heraeus™ Megafuge™ 16, Thermo Scientific™, Germany) to remove the biomass. Afterwards the crude laccase extract was vacuum filtrated through a 0.7-µm glass microfibres filter grade 698 (VWR International, Darmstadt, Germany). A higher laccase concentration of the permeate was achieved using crossflow filtration cassettes (Vivaflow^®^ 50 R, Sartorius, Göttingen, Germany) made of polyethersulphone (PES) with a molecular weight cut-off (MWCO) 10 and 30 kDa at 22 °C. Flow rate was adjusted between 100 and 200 mL/min using a peristaltic pump (Masterflex L/S^®^, Cole-Parmer, Wertheim, Germany). Retentates were frozen at −80 °C overnight, followed by freeze-drying for 24 h (Christ, Osterode, Germany). Samples were stored at 4 °C.

Flax fibres were exposed to laccase treatment with different methods, depending on the later investigations. For the tensile test investigation of fibre, flax yarn was loosely wrapped around a short rod which was submerged in malonate buffer with 25 U/mL laccase for 2 h. For the micromechanical investigations, single fibres were submerged in malonate buffer with 3.3 U/mL laccase and 5 mg dopamine for 2 h. The modifications were performed using a Thermomixer (Eppendorf, Hamburg, Germany) with 600 rpm at 25 and 50 °C. Previous experiments have shown laccase is very stable up to temperatures of <60 °C by a residence time of more than 1 h. After treatment, the fibres were washed in water and dried at 40 °C.

### 2.2. Fibre Tensile Test

The longitudinal tensile strength of single flax fibres was determined in a Favigraph single fibre tester (Textechno, Mönchengladbach, Germany). The fineness was determined vibrationally for each individual fibre. Consecutively, the single fibre’s force elongation curve was measured. From this, the Young’s modulus (0.05% to 0.5% elongation) and strength were determined. The measurement was performed with 25 repetitions at 15 mm/min with fibres clamped between vulcanite fasteners with 6 bar of pressure.

### 2.3. Fibre Morphology

The fibre surface was investigated by digital microscopy with a digital microscope VHX-100 (Keyence, Osaka, Japan) and by scanning electron microscopy (SEM) with the scanning electron microscope FE-SEM Ultra 55 (Carl Zeiss SMT AG, Oberkochen, Germany) with an Everhart–Thornley detector. AFM measurements were carried out by a Dimension nanoscope (Bruker-Nano, USA) in the tapping mode under ambient conditions (ScanAsyst fluid + cantilevers, Bruker-Nano, Billerica, MA, USA).

### 2.4. Single Fibre Pullout Test

Single fibre pullout tests were performed on a device developed and set up at the IPF [45], 8 to 10 samples were studied for each modification. A micro-composite was produced by embedding one end of a flax fibre into a droplet of epoxy RIM 135 matrix (curing for 60 min at 85 °C in nitrogen atmosphere). The embedding length was preset to 100 µm. 

The vessel containing this micro-composite was clamped on an actuator. The fibre end protruding out of the matrix was glued onto a steel plate mandrel which was fixed to a force sensor. Force–displacement curves were obtained by quasi-statically pulling the single fibre out of the matrix droplet. One distinctive feature of the device is the extremely low pullout speed, the presented data were obtained at 10 nm/s during interphase fracture and 1µm/s in the friction phase. Forces between 1 mN and 5 N can be detected by a load cell. 

After pullout, the fibre diameter and the embedding length (tip to meniscus) were checked for each fibre by SEM. Diameters were in the range of 20 ± 5 µm, the embedding lengths were in the range of 90 ± 15 µm.

## 3. Results and Discussion

**Figure 1:**
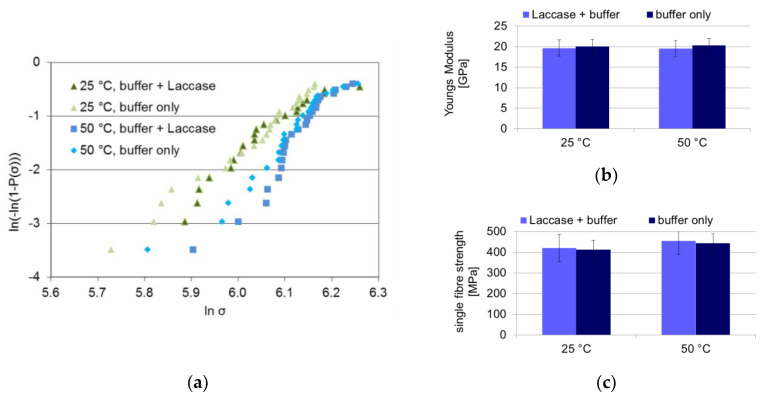
Results of single fibre tensile tests of buffer- and laccase-treated fibres and buffer-only fibres (“control”) for 25 and 50 °C: (**a**) Weibull plot, (**b**) Young’s modulus, (**c**) tensile strength. In (**b**,**c**), the mean and an error of one standard deviation is shown.

The aim of the study is to use the laccase enzyme to bind a dopamine coupling agent onto the lignin contained within the fibre surface. However, the laccase should not compromise the fibre strength during the treatment, e.g., by degrading the cellulose. In order to ensure that the laccase does not deteriorate the celluloses properties during lignin modification, fibres were laccase-treated at 25 °C and for an enhanced effect at 50 °C, at an enzyme activity eight times higher than that used for dopamine binding in order to rule out any fibre deterioration. The tensile strength of the treated fibres was measured in a 25-fold measurement. The resulting Weibull plot as well as mean single fibre tensile strength and Young’s modulus are shown in Figure 1.

The Weibull distributions of the laccase-treated and control fibres coincide for both 25 and 50 °C, respectively. The fibre fracture characteristics are not modified by the laccase treatment, not even at the high laccase concentration used. Within the error range, no decrease in mean tensile strength is detected, not even at higher temperatures. The fibre Young’s modulus is not affected by the laccase treatment, either. Evidently no degradation of cellulose occurred during the laccase treatment under the current conditions.

As stated in the introduction, a modification of flax fibres with laccase alone in order to improve covalent bonding to epoxy matrices is feasible. However, in this case the fibre matrix linkage process is limited to the accessibility of functional groups due to steric hindrance. Besides, according to Grönqvist et al., the induced radicals necessary for coupling reactions are short-lived and coupling to matrix molecules would need to be performed immediately after the lignin modification [42,46]. Better results are expected if a bi-functional coupling agent is used. This coupling agent should covalently bind to the fibre during fibre modification and to the epoxy matrix during composite formation. 

**Figure 2:**
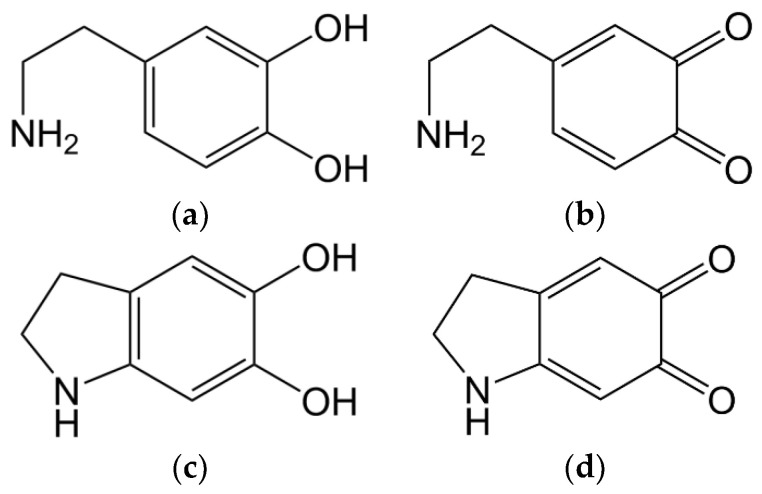
Formulas of chemical compounds representing intermediate steps of laccase-catalysed oxidation of dopamine (**a**) via dopamine-o-quinone (**b**) and 5,6-dihydroxyindoline (**c**), forming indole-5,6-quinone (**d**).

Dopamine and other amino phenols have been investigated as laccase substrates before [42,43,46]. When dopamine (Figure 2a) is oxidised by laccase on its own, phenol groups get oxidised, resulting in a quinone structure (Figure 2b). These structures are highly reactive and intramolecular Michael-additions can occur (Figure 2c), followed by further laccase-catalysed oxidation, forming indole-5,6-quinone (Figure 2d).

**Figure 3:**
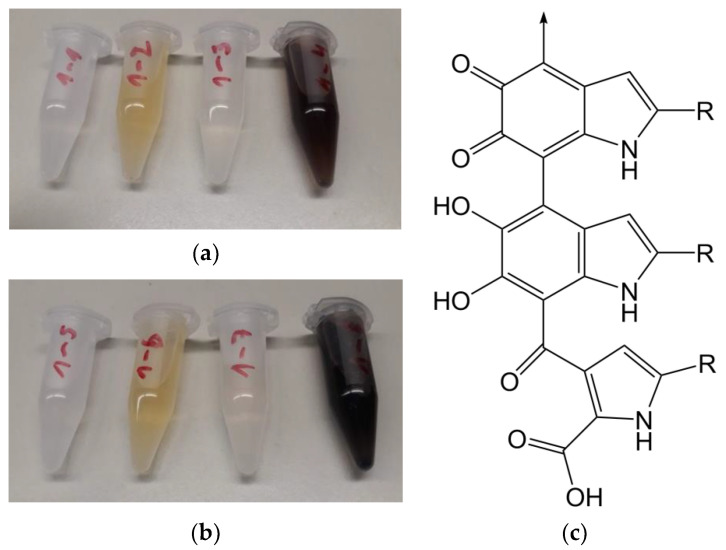
Modification and control solutions (**a**) 25 °C and (**b**) 50 °C. From left to right: buffer, buffer + laccase, buffer + dopamine, buffer + laccase + dopamine, each modified on a shaker at 600 rpm for 4 h without immersed fibre. (**c**) Structure of eumelanin, a substructure of natural melanin.

Fibres were exposed to laccase at 3.3 U/mL in malonate buffer at 25 and 50 °C, together with dopamine. The buffer stabilises the enzyme and ensures consistent enzyme activity. The combination of dopamine and laccase, with or without immersing a fibre, showed a pronounced colour change, turning the solution dark brown. A brown colour is also visible on the dopamine- and laccase-treated fibres. The modification and control solutions are shown in Figure 3a,b.

**Figure 4:**
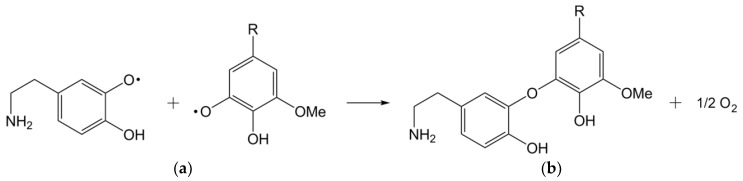
Bonding of (**a**) dopamine to (**b**) lignin via radical coupling.

Different possibilities for the lignin and dopamine coupling reaction have been discussed. One possibility is a radical coupling between phenoxy radicals on both lignin and dopamine. Evidence for this was found by Grönqvist et al. [42] in the form of ether linkages between laccase-modified lignin and modified tyramine, a molecule very similar to dopamine. This would potentially leave an accessible primary amine group which in turn could bond covalently to polymer matrices in subsequent processing steps. Another possibility for coupling dopamine to lignin is over a quinone-like species, which can be formed by laccase-mediated radical oxidation which undergoes a reaction with the amine group of the dopamine [46]. However, subsequently, an intramolecular Michael-addition of dopamine on itself [43] is expected. Therefore, the formed secondary amine is less reactive due to the lower steric accessibility. Hence, for a polymer matrix coupling, only the phenol groups of dopamine are available. Moreover, the intramolecular Michael-addition products, like e.g., indole-5,6-quinone, are able to polymerise to polydopamine, this can be further accelerated by laccase catalysis. These are supported by Li et al. [46], they reported that different monomers like indole-5,6-quinone and 5,6-dihydroxyindole can be polymerised via an ether-linkages into polydopamine. A comparable natural substance molecule is the brown pigment eumelanin, a substructure of natural melanin, pictured in Figure 3c. Such structures could be responsible for the dark brown colour of the modified fibres and the modification solution. When both dopamine and lignin are oxidised with laccase-catalysation, covalent coupling reactions between both compounds are feasible (Figure 4).

**Figure 5:**
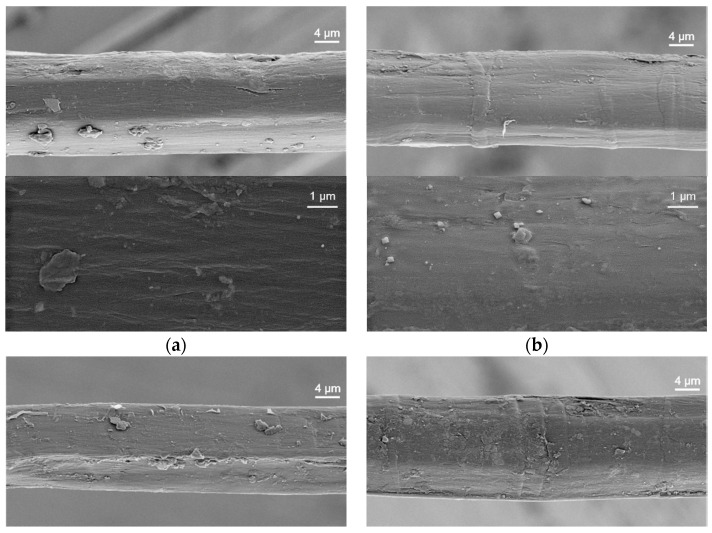

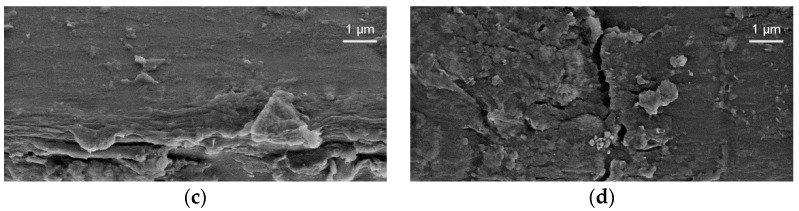
SEM images of two magnifications of flax fibres: (**a**) buffer, (**b**) buffer + laccase, (**c**) buffer + dopamine, (**d**) buffer + laccase + dopamine, after modification at 25 °C in each case.

**Figure 6:**
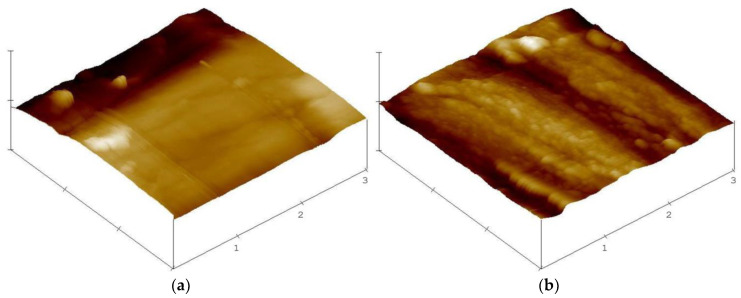
AFM images of flax fibres: (**a**): buffer only, (**b**): buffer + laccase + dopamine. Height and length scale 1 µm.

Scanning electron microscopy (SEM) was performed in order to investigate the change in surface morphology after treatment with buffer only, buffer + laccase, buffer + dopamine, buffer + laccase + dopamine. The micrographs are shown in Figure 5. When the fibre is modified with the buffer only or laccase (Figure 5a,b), the surface is smooth, compared to a modification with dopamine or laccase + dopamine (Figure 5c,d), where the surface is covered by a flake layer. If laccase is used to catalyse the process, the flakiness is more pronounced. We propose to interpret the formed structures as a polydopamine layer. As dopamine can polymerise on its own, it is feasible that polydopamine will form on the surface even in absence of laccase. If laccase is present, the polydopamine layer is enhanced, leading to the fibre colour change to a darker brown. This flaky layer covering the flax surface is also verified by AFM micrographs of buffer + laccase + dopamine-treated fibre as compared to the fibre treated with buffer only (Figure 6).

The aim of the fibre modification is to improve fibre matrix adhesion strength. To quantify how the fibre will perform in fibre-reinforced composites, micro-mechanical tests were performed on single fibre model composites, namely single fragmentation tests (SFFT) and single fibre pullout test (SFPT).

Single fibre fragmentation tests were performed on flax fibres treated in a laccase + dopamine + buffer as compared to reference treatments (water, buffer only, laccase + buffer, dopamine + buffer). Because of the low elongation of the matrix and the flax fibres used in this study, the fragmentation test results scatter strongly and provide no significant result. However, a tendency towards a higher number of fibre fractures is visible for fibres modified with dopamine, and more so for fibres modified with laccase and dopamine (cf. Supplementary Materials: Figures S1 and S2). A higher number of fractures is related to a higher surface strength. Dopamine, and more so laccase-dopamine treatment, seems to improve interfacial properties.

Single fibre pullout tests ensure more precise measurements, which yield quantitative values for the interfacial shear strength of a fibre matrix interphase in a single fibre model composite. Because of the high effort of single fibre pullout tests, only samples treated in buffer + laccase + dopamine and control samples treated in buffer were measured. 

**Figure 7:**
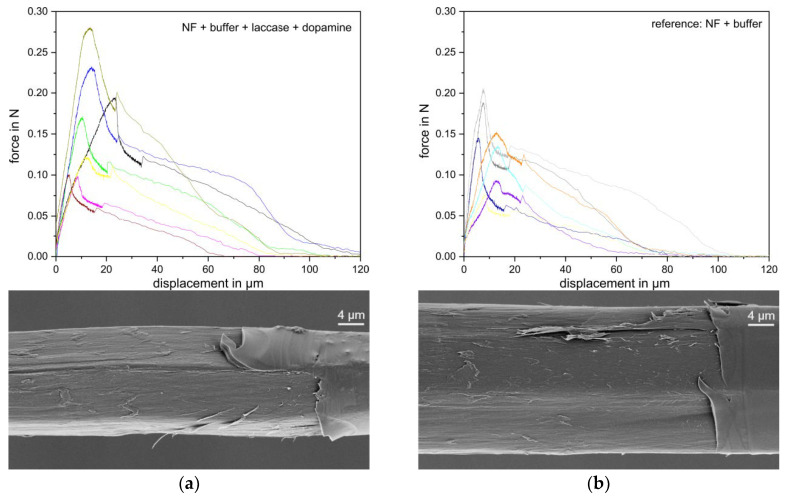
Top: single fibre pullout data of (**a**) laccase+dopamine-treated flax fibres in epoxy matrix and (**b**) buffer only-treated fibres in epoxy matrices; bottom: SEM images of respective fibre after pullout. Each SEM micrograph shows the original fibre surface on the image right edge, and the exposed fracture surface of the fibre after pullout on the left and centre of the image.

Force–distance curves of the pullout process of single fibre microcomposites have a characteristic shape. During the pullout test, the force–distance curves initially rise steeply (see Figure 7 for this study’s curves), and in this phase the interphase is intact. At some point, interphase failure starts with slow crack propagation, but the remaining intact part of the interphase is still able to bear load up to the total interphase failure, at the force maximum. This is followed by a friction-dominated pullout of the fibre from the former interphase tunnel. The pullout process was investigated profoundly by Zhandarov et al. [47,48,49].

The embedded lengths seen from pullout and from microscopic analysis of the pulled out fibre, were in the range of 0–120 µm, meaning several fibres failed during the pullout measurement, either in the gap between the pulling mandrel and the matrix droplet surface, or often just below the surface. Besides, some fibres fractured lengthwise, leaving a long and pointed tip with lower friction or a frayed tip, or fibres broke in a series of sub-fractures. Data sets with either of these two atypical fracture types are clearly discerned from standard data sets in both force–distance curves (no friction behaviour) and upon microscopic control of the pulled out fibre (length tip to meniscus < 70 µm) and they were omitted in the evaluation.

**Figure 8:**
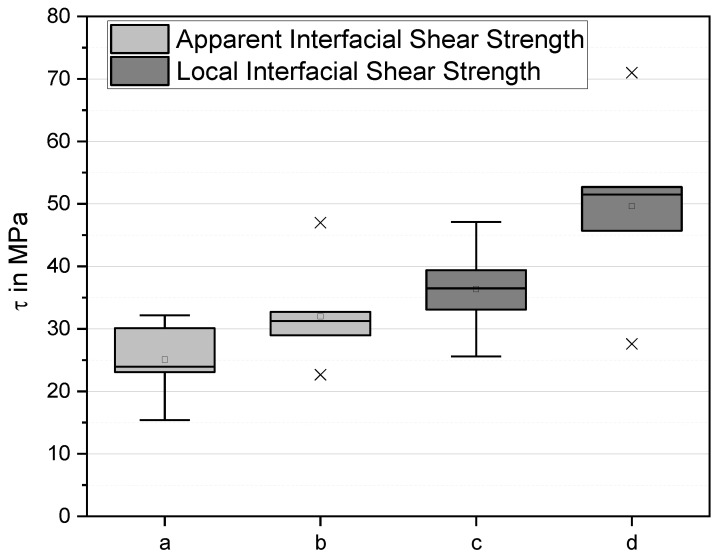
(a,b) Apparent interfacial shear strength (IFSS) (τ_app_) and (c,d) local IFSS (τ*_d_*), 7 evaluable samples each, modification with buffer only (a,c) and buffer + laccase + dopamine (b,d). Results are presented in a boxplot format, the horizontal line mark the median, the hollow dot marks the mean, boxes represent the centre quartiles, whiskers denote 1.5 times the interquartile range, × marks outliers.

The force curves vary in amplitude, in accordance with their interfacial area (Figure 7). They coincide much more when normalised by the individual fibres mantle area A= 2π r l, with *r* fibre radius and *l* embedded length (determined post-pullout in a SEM micrograph). The force curves are evaluated in two different manners, highlighting different aspects of the fracture behaviour. The apparent shear strengths are calculated as τ_app_ *= F_max_/2πrl = F_max_/A* with *F_max_* the maximum force. They characterise the maximum load the interphase is able to bear. This is a parameter relevant in practice. However, it is nonlocal, averaged over fibre regions of strongly differing local shear stress. The apparent shear strength values are τ_app_ = (34 ± 6) MPa for the laccase- and dopamine-treated fibre compared to τ_app_ = (24 ± 6) MPa for the untreated fibre (Figure 8), i.e., the apparent IFSS increases by 30%.

We also used the experimental force–displacement curves to calculate the *local* interfacial shear strength, IFSS or τ*_d_*, determined at maximum interfacial stress near the tip of a crack propagating along the fibre [50]. In this approach, the crack length, *a*, was expressed as a function of the force, *F*, applied to the fibre, with local interfacial shear strength τ*_d_* and the interfacial frictional stress, τ*_f_*, as parameters to be determined from experimental data, as suggested by Zhandarov et al. [47,48]. This evaluation is more precise than that using the debond force *F_d._* [47].

The results are shown as box plot in Figure 8. The boxes denote the quartiles, the whiskers extending from the boxes indicate variability outside the upper and lower quartiles, outliers are plotted as individual x. As can be seen, the local IFSS τ*_d_* increases upon treatment by about 30% from (38 ± 8) MPa to (50 ± 10) MPa. The critical energy release rate G_ic_ also increases in trend, from (2.7 ± 2) J/m² to (3.4 ± 2) J/m². It is interesting that the determined interfacial frictional stress, τ*_f_*, also increased, by 35% from (20 ± 6) MPa to (27 ± 6) MPa. In all probability, this is due to the fact that similar mechanisms (molecular forces and interface roughness) are responsible for interfacial adhesion and friction between fibre and matrix. 

Increasing the roughness of a fibre surface has been reported to be a physical method of improving the interfacial strength [50]. This can occur either by increasing the contact area and therefore the total surface energy or by mechanical interlocking. In either case, the roughness must be on a firmly bound surface and not on the surface of a soft, non-cohesive layer. Comparing the surface area of the four fibre types on the 5-µm scale, the contact area is comparable. The surface of the fibres seen in Figure 5 is still dominated by the polygonal cross section typical of the flax fibre. On the 100 nm scale seen by AFM, the microscopic surface area may be increased somewhat. It is feasible that the newly formed, microscopic surface structures lead to a slight microscopic interlocking compared to the original fibre shape. However, if there is chemical bonding, the effect of the covalent bonds usually surpasses that of physical interaction.

SFPT is a very interphase sensitive method, as during pullout, the fracture occurs along the fibre matrix interface. Figure 7 shows electron microscopic images of the fracture surface as seen on the fibres after they have been pulled out the micro composite. Note this is not the fibre surface seen in Figure 5. Either type of pulled out fibre show an angular surface pattern. Such a pattern has been reported to relate to the exposed cellulose fibrils, indicating that the outer fibre surface is partially sheared off [10].

**Figure 9:**
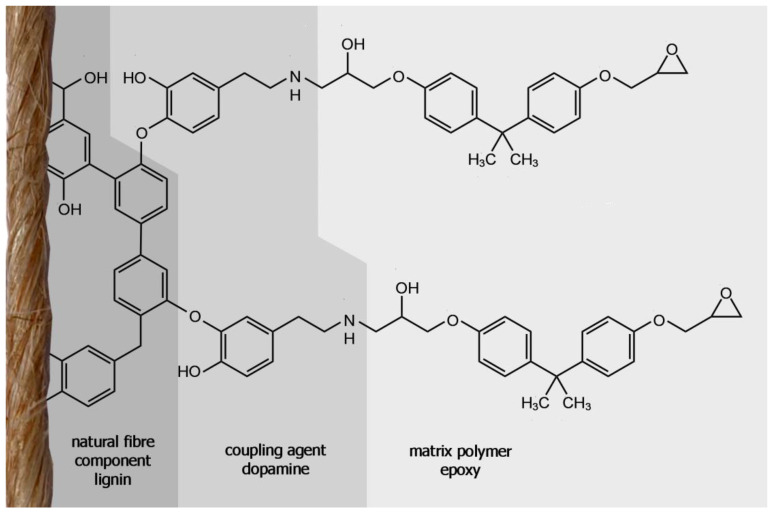
Presumed chemical structure between flax fibre lignin and epoxy matrix polymer by laccase-induced coupling of dopamine.

The laccase treatment with dopamine as a coupling agent has enhanced the adhesion between fibre and matrix presumably by bonding the dopamine to the lignin in the flax fibre which can then in turn link to the epoxy matrix. Because the amine groups from dopamine have a higher reactivity at the fibre surface compared to hydroxyl groups from cellulose or lignin, the epoxy can now covalently bond to the fibre more easily, if amine-curing epoxies are used as matrix. A model for this coupling is presented in Figure 9.

## 4. Conclusions

Natural fibre composites have a low ecological footprint. For conventional fibres, the use of surface modification such as sizing or finish is the state-of-the-art method. However, the agents used in these modifications are often not ecological. The study presents a sustainable alternative for natural fibres: an enzymatic treatment using the naturally abundant enzyme laccase with the biological reagent dopamine as a coupling agent. Laccase is a fungal enzyme that can infer an oxidation in lignin and thereby bond substrates as coupling agents to natural fibres. If these coupling agents are bifunctional such as dopamine, they have the potential to establish a covalent bond to a corresponding matrix.

In nature, laccase is part of a fungal enzyme cocktail that degrades wood and cellulosic materials. In an essential control experiment it was checked that the flax fibres are not damaged by the laccase at the conditions harsher than those used in this study: the tensile strength and Young’s modulus of the fibres remain constant during treatment. Laccase and dopamine treatments deposit an additional layer on the fibre surface, as seen in a brownish hue on the fibre, in SEM and AFM micrographs of the fibre surface. 

Enzymatic modification of natural fibres in order to improve wetting has been successfully carried out before [33,34]. The present study shows that the approach will also go beyond improved wetting: laccase will covalently bond a reactive layer, proposedly consisting of coupling agent, to the surface. In this way, a covalent bond from fibre to matrix can be formed. This improves the interfacial shear strength of a natural fibre epoxy microcomposite by 30%. The use of sustainable materials together with a sustainable modification technique paves the way for fully “green” composites.

## Supplementary Materials

The following are available online at https://www.mdpi.com/1996-1944/13/20/4529/s1, Figure S1: Example light microscopy micrograph under polarization slide for fragmentation tests with 3 visible fibre fractures, Figure S2: Fracture Counts for Single Fibre Fragmentation Test (SFFT). (a): Reference in water, (b): buffer, (c): buffer + laccase, (d): buffer + dopamine, (e): buffer + laccase + dopamine; n = 16.
