# Supplementary material for: Laccase-Enzyme Treated Flax Fibre for Use in Natural Fibre Epoxy Composites
Source: Materials (Basel). 2020 Oct 13;13(20):4529. doi: 10.3390/ma13204529 (PMC7600163; doi:10.3390/ma13204529)
Supplement: Supplementary file 1 [file materials-13-04529-s001.pdf]

materials

Article

Laccase-Enzyme Treated Flax Fibre for Use in Natural Fibre Epoxy Composites

Hanna M. Brodowsky [1,*], Anne Hennig [2], Michael Thomas Müller [3], Anett Werner [4], Serge Zhandarov [5] and Uwe Gohs [6]

Received: 29 August 2020; Accepted: 5 October 2020; Published: date

Supplementary information

Single Fibre Fragmentation Tests

For single fibre fragmentation tests, specimens consisting of a matrix with a single embedded fibre are subjected to a longitudinal tensile test. As the matrix is elongated, the interphase is exposed to shear stress. The fibre fails within the specimen before total specimen failure occurs. The resulting fragment length is inversely related to the interfacial shear strength: a low mean fragment length or high number of fractures indicates high interphase strength.

Individual 4-cm fibres were obtained from Setral Chemie, (Germany), and cleaned in the Setralit® process. They were modified as described above, but with 1 U/ml laccase and 5 mg dopamine for 5 h in malonate. The single flax fibres are embedded in epoxy L 20 + EPH 161 and in a second round of experiments in L+L, both obtained from R&G Faserverbundwerkstoffe GmbH, Germany. The composites are cured at room temperature for 24 hours and tempered at 60 °C for 10 hours. Dumbbell-shaped test specimens of 2 cm in length were cut with one single fibre oriented along the long axis. Tensile tests with 0.2 mm/min are performed up to specimen failure. Resulting fibre fractures are characterized with transmission microscopy (Keyence VHX-2000) using a polarizing slide. Fibre fracture is visible as a darkened part of the fibre and by the V- or X-shaped patterns of polarized light indicating internal stress in the surrounding matrix (exemplified in Figure Supplement 1). The amount of fibre fractures for each modification process was calculated as the mean from 16 microcomposites, each analysed over the gauge length of about 1 cm. The result is shown in Figure Supplement 2.

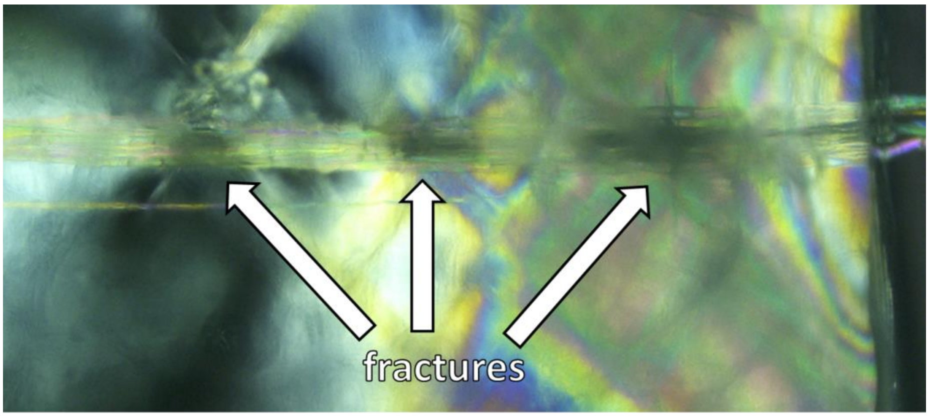

Figure S1. Example light microscopy micrograph under polarization slide for fragmentation tests with 3 visible fibre fractures. Right hand side: composite failure with protruding fibre.

As a rule of thumb for single fibre fragmentation tests, the fibre elongation should be at least 2.5 times lower than the matrix elongation to ensure fibre fractures before matrix damage. Because of the low elongation of the epoxy matrix used in this study, the fragmentation test results scatter widely. However, a tendency towards higher amounts of fibre fractures is visible as the fibres

become modified with dopamine and laccase+dopamine. Both the water-treated and buffer only-treated reference fibres show fewer fibre fractures. The addition of only laccase shows lower amounts of fractures than those with additional dopamine, presumably because no coupling molecule was added and therefore no assisted linkage between flax fibre and epoxy matrix occurred. Dopamine without laccase increases the number of fractures. This might be due to the fact that dopamine can oxidise and therefore bind to the fibre on its own. This step is catalysed and drastically accelerated by laccase.

A modification at 50 °C was tested as well, showing overall higher amounts of fibre fractures than for fibres treated at 25 °C. The amount does not vary in accordance with the laccase / dopamine concentrations in the solution and the difference cannot be attributed to higher laccase activity, because even the buffer-only solution shows high amounts of fibre fractures. Therefore, the effect can be attributed to the temperature increase itself. For evaluation, only fibres treated at 25 °C were considered. This ensures a clear result towards the influence of the laccase/dopamine treatment on the adhesion between fibre and matrix.

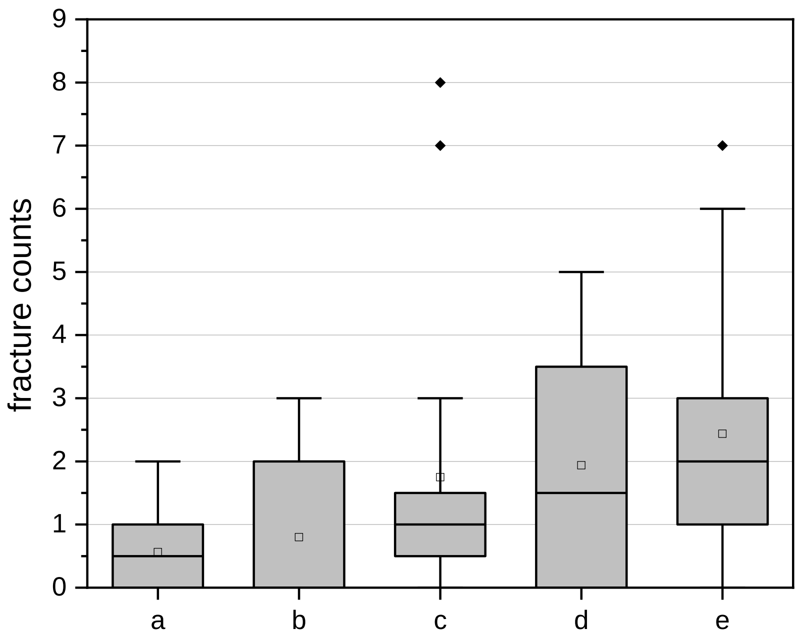

Figure S2. Fracture Counts for Single Fibre Fragmentation Test (SFFT). a): Reference in water, b): buffer, c): buffer + laccase, d): buffer + dopamine, e): buffer + laccase + dopamine; n = 16.

© 2020 by the authors. Submitted for possible open access publication under the terms and conditions of the Creative Commons Attribution (CC BY) license (http://creativecommons.org/licenses/by/4.0/).
